# Dietary changes and anxiety during the coronavirus pandemic: a multinational survey

**DOI:** 10.1038/s41430-021-00897-3

**Published:** 2021-03-19

**Authors:** Vered Kaufman-Shriqui, Daniela Abigail Navarro, Olga Raz, Mona Boaz

**Affiliations:** 1 Department of Nutrition Sciences, Ariel University, Ariel, Israel; 2 Centre for Urban Health Solutions (C-UHS), St. Michael’s Hospital, Toronto, ON Canada

**Keywords:** Risk factors, Infectious diseases

## Abstract

**Background/objectives:**

The 2020 global coronavirus pandemic is characterized by increased anxiety. Anxiety has been associated with poor diet quality and weight gain, which may lead to obesity, a risk factor for adverse COVID-19 outcomes. The present study was designed to examine associations between diet quality and anxiety levels during the COVID-19 pandemic.

**Subjects/methods:**

This cross-sectional, international online study was conducted between March 30 and April 25, 2020 and available in seven languages: Arabic (7.6%), English (43.7%), French (0.8%), Hebrew (42.1%), Italian (3%), Russian (1.1%), and Spanish (1.6%). Diet quality was assessed using the Mediterranean Diet Score (possible range: 0–17 points) and anxiety scored using the General Anxiety Disorder 7-point scale (GAD-7). The Google Survey platform was used to conduct the survey.

**Results:**

A total of 3797 persons were included in the present analysis. More than 75% of respondents were female; most completed the survey in English or Hebrew. Median age was 31 (IQ = 18) years. Almost 60% indicated that their pre-pandemic diet was healthier than their current diet. The median Mediterranean diet score was 9 (IQ = 3). The majority (54%) of participants reported at least mild anxiety, while 25% reported moderate anxiety or more severe. In a logistic regression model of at least moderate anxiety, Mediterranean diet score (OR 0.92, 95% CI 0.89–0.95, *p* < 0.0001) reduced odds of elevated anxiety, even after controlling for age, sex and other variables.

**Conclusions:**

Though causality cannot be inferred, associations between diet quality and anxiety might suggest public health interventions including diet and stress control during future mass lockdowns.

## Introduction

The global SARS-CoV-2 COVID-19 (coronavirus) pandemic outbreak has prompted many governments to enforce strategies of screening, mitigation, wide-scale self-isolation, and/or suppression to control the outbreak [1, 2]. The outbreak itself and the measures taken to control it have been associated with anxiety [3, 4].

An association between anxiety and intake of specific foods has been documented. Animal studies demonstrate that intake of sweet and palatable foods appears to confer stress relief while the withdrawal of long term excessive lipid intake increases stress [5]. In humans, elevated intake of added sugars and saturated fats, but not increased calorie intake per se, has been shown to be positively associated with anxiety [6]. More generally, anxiety has been associated with obesity and other risk-to-health behaviors in adults [7]. This is relevant in the context of the COVID-19 pandemic because 94% of hospitalized COVID-19 patients who have died had at least one obesity-associated comorbidity such as diabetes, cardiovascular disease, chronic lung disease, hypertension, and certain cancers [5–9]. Conversely, a healthy diet may protect against anxiety [8].

The Mediterranean diet (MedDiet), characterized by frequent intake of olive oil, fruits, vegetables, whole grains, legumes, fish and nuts, and infrequent intake of red and processed meats and added sugars, is associated with reduced all-cause and cause-specific mortality as well as reduced risk of chronic degenerative diseases including certain cancers, type 2 diabetes and cardiovascular disease [9–12]. The MedDiet score has also been shown to be inversely associated with depression and anxiety among adults [13]. Thus, the degree to which an individual diet is similar to the MedDiet can be used as an estimate of diet quality [14].

It was thus hypothesized that diet quality would be associated with anxiety during the COVID-19 2020 pandemic.

## Objectives

The present study was designed to estimate the degree to which dietary habits were consistent with the MedDiet pattern, which has been associated with reduced all-cause mortality [15] and to determine the degree to which adherence to the MedDiet pattern is associated with anxiety [16] during the 2020 COVID-19 pandemic.

## Methods

### Study design

The present cross-sectional survey was conducted online using a convenience sample. The purpose of the convenience sample was to permit immediate access to as many respondents as possible during the acute lockdown period of the pandemic. The survey simultaneously assessed the degree to which the current participant diet is similar to the MedDiet; the degree to which the MedDiet score was associated with anxiety; the degree to which the current diet was similar to the diet before the pandemic; and demographic characteristics.

### Ethics

The study was approved by the Institutional Ethics Board (Helsinki Committee) of Ariel University, Israel. Each participant provided informed consent prior to responding to the survey. Individuals who did not provide informed consent (indicated by clicking on the appropriate button) could not proceed with the survey.

### Study location

The study was conducted online using a Google Survey platform. The survey was uploaded to several social media sites, including but not limited to the Ariel University Department of Nutrition FaceBook page; and the r/Coronavirus community on reddit, which created a page for pandemic-associated research; and individual social media pages. Participants were encouraged to re-post the survey link on their own social media in order to expand exposure.

### Study population

The study population included all adult individuals who elected to complete the survey online and who provided informed consent by clicking on the appropriate button.

### Inclusion criteria

All adult individuals (aged 18 or older) who saw and elected to respond to the survey were included in any of the study languages: Arabic, English, French, Hebrew, Italian, Spanish or Russian.

### Exclusion criteria

Subjects who indicated that their age is younger than 18 years, and those who did not provide informed consent were excluded.

### Study procedures

The survey was posted to public and personal social media pages. Individuals could respond after providing informed consent. Responses were automatically recorded in Google Survey.

### Data acquisition and survey characteristics

The following data were elicited in the survey: (1) demographic information: age; sex; country of residence; education; work setting; current occupational status; current quarantine/isolation; and health status; (2) nutrition questionnaire; and (3) anxiety questionnaire. Data were anonymous, though respondents had the option of providing an e-mail address to which information about his/her MedDiet score was mailed. The survey was translated from its original Hebrew to the following languages: Arabic, English, French, Italian, Russian, and Spanish. Each translation was performed by a native speaker of the target language who was also fluent in Hebrew. Once translated, the translation was back-translated to Hebrew by an individual bilingual in both the target language and Hebrew, thus validating the translation.

### MedDiet score

Scores for evaluating the degree to which an individual diet is similar to the MedDiet have been developed [9, 17, 18]. Several shorter screeners have been validated as tools for rapid assessment of MedDiet adherence for risk assessment [9, 17, 19]. The Israeli Mediterranean diet screener (I-MEDAS) used in the current study was not intended to capture the dietary nuances of study participants, who were from six different continents; rather, the score was intended to approximate diet quality regardless of cultural framework. It was adapted from the original, 14-item questionnaire and modified to make it applicable also to the Israeli population [15]. The I-MEDAS is scored such that a given question receives a value of 1 if the criterion was met, and 0 if it was not, so the total I-MEDAS score can range from 0 to 17 points [15].

### Anxiety score

The 7-item Generalized Anxiety Disorder Scale (GAD-7) was used to measure anxiety. The GAD-7 is a self-reported anxiety questionnaire validated for use in the general population, [20] and asks respondents to refer to the 2 weeks prior to the survey when responding to a set of statements. Each item on the scale can receive a score as follows: 0 (not at all), 1 (several days), 2 (more than half of the days) 3 (nearly every day); thus, the total score can receive value from 0 to 21, where a higher score indicates greater anxiety [21]. Cut-off scores for mild, moderate and severe anxiety symptoms are 5, 10 and 15, respectively [22]. The GAD-7 was already validated for use in the languages in which the present survey was conducted, so further translation was not necessary.

### Sampling procedures

The survey was uploaded to both public and personal social media sites, with a request to respondents to re-post the survey. Thus, the sampling method is best described as a convenience sample with a snow-ball distribution.

### Statistical analysis

Data were downloaded from Google Survey to Excel (Microsoft USA) and analyzed on SPSS v25 Statistical Analysis Software (IBM Inc.). Distributions of continuous variables were assessed for normality using the Kolmogorov–Smirnov test. All continuous data had distributions significantly deviating from normal, so are described as median (interquartile range). Nominal variables were presented as *n* (%). Associations between continuous variables were described by calculating the Spearman’s correlation coefficient. Continuous variables were compared by nominal variables using the Mann–Whitney U test or the Kruskal–Wallis test as appropriate. Associations between nominal variables were assessed using the chi-square test. The Wilcoxon signed ranks test was used to compare those measures for which a value before vs. after the coronavirus pandemic were available. A multivariable logistic regression model was developed to identify the cut-point for moderate anxiety or greater (GAD-7 score ≥ 10) [21, 22] into which variables by which GAD-7 differed in univariate analysis were entered. The final model was arrived at using a backward stepwise approach with the likelihood ratio test, with an entry probability of 0.05 and a removal probability of 0.1. Odds ratios with 95% confidence intervals were calculated for each covariate, and a classification table was created. All analyses were two-sided and considered significant at *p* < 0.0001.

### Sample size and study power

With 2144 anticipated responses, the study was powered to provide a 95% confidence level and a confidence limit of 2% for a GAD score of 10 or greater. The calculated sample size also provided 99% power to detect a true association between the GAD score and MedDiet score of *r* = 0.15 or greater, with alpha = 0.0001.

## Results

### Database and participant dispensation

**Fig. 1:**
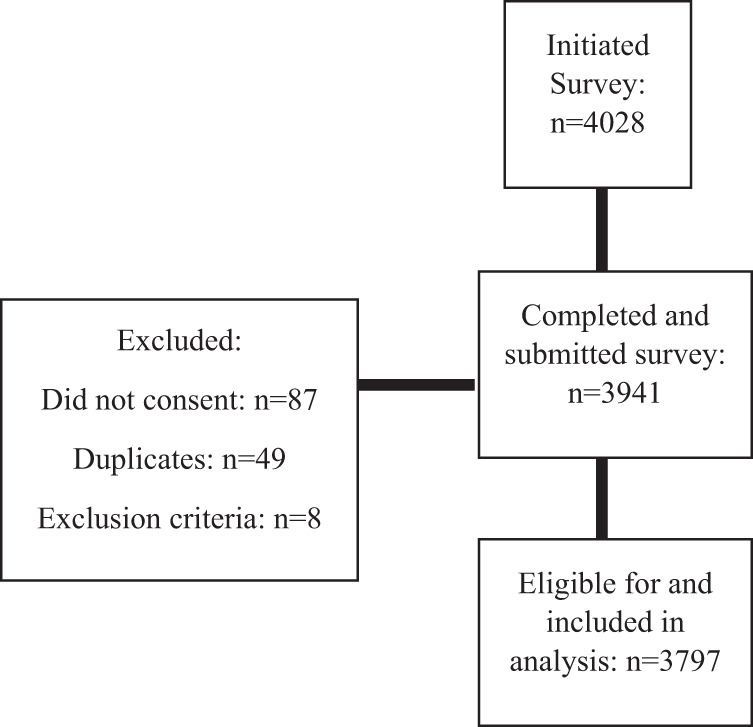
Patient dispensation. The flow of participants in the present survey. As can be seen, a total of 4028 individuals initiated the survey, and 3941 (97.8%) submitted a completed survey. Of these, a total of 144 (3.64%) were excluded from the present analysis due to the following reasons: 87 did not provide informed consent; 49 surveys were identified as duplicate responses from individuals who had already submitted a survey (the first survey was included and the subsequent survey was excluded); and 8 met exclusion criteria—specifically, age <18 years. A total of 3797 participants were included in the final analysis.

On April 25 22:00 Israel time, the questionnaire was closed to responses and the database was downloaded. Participant dispensation is presented in Fig. 1. Of the 4028 individuals who initiated the survey, 3941 completed and submitted it. Omitted were 49 duplicate responses and eight respondents who indicated age younger than 18 years. A total of 3797 were included in the analysis.

### Participant characteristics

Characteristics of the study population are presented in Table 1. Of the 3797 participants in the present survey, more than 75% were female. The majority of respondents answered the survey in English or Hebrew. Most participants were non-smokers, currently employed and presently working, and had a bachelor’s degree. Only three respondents had a confirmed coronavirus diagnosis at the time of completing the survey, but 79 were suspected cases without laboratory confirmation. Another 16 were recovering from the virus. Of the 2854 female participants, few were pregnant or within 6 months of delivery.

**Table 1:** Study Population Characteristics.

Characteristic	Population value
Age (years, median (interquartile range))	31 (18)
Sex *n* (% female)	2854 (75.2)
Reproductive status (Females only) *n* (%)	
Neither pregnant nor 6 months post delivery	2328 (81.6)
6 months or less post delivery	75 (2.6)
Currently pregnant	42 (1.5)
Language *n* (%)	
Arabic	287 (7.6)
English	1661 (43.7)
French	31 (0.8)
Hebrew	1600 (42.1)
Italian	114 (3.0)
Russian	43 (1.1)
Spanish	61 (1.6)
Health Status *n* (%)	
Healthy	3655 (96.3)
Sick with coronavirus	3 (0.1)
Suspected of coronavirus	79 (2.1)
Recovering from coronavirus	16 (0.4)
Sick but not with coronavirus	44 (1.2)
Smoking status *n* (%)	
Never smoked	2432 (64.0)
Former smoker	845 (22.3)
Occasional smoker	346 (9.1)
Smokes at least one cigarette/day	174 (4.6)
Usual Place of employment *n* (%)	
Large/medium private sector company	822 (21.6)
Small private sector company	527 (13.9)
Public sector/non-profit	1162 (30.6)
Pensioner/unemployed	825 (21.7)
Independent business owner/freelancer	439 (11.6)
Other	22 (0.6)
Work status during pandemic *n* (%)	
Leave of absence with pay	260 (6.8)
Leave of absence without pay	661 (17.4)
Retired/pensioner	89 (2.3)
Still working	1890 (49.8)
Unemployed	658 (17.3)
Other	239 (6.3)
Education *n* (%)	
Fewer than 12 years	54 (1.4)
High school diploma/matriculation	925 (24.4)
Professional license (technician, tradesperson, etc.)	290 (7.6)
Bachelor’s degree	1412 (37.2)
Master’s degree or higher	1096 (28.9)
Other	20 (0.5)

### Lifestyle and dietary characteristics

Table 2 displays the lifestyle and dietary characteristics of the study population. The median MedDiet score was 9 (3) in the study population. The number of servings for each of the items queried is also displayed.

**Table 2:** Lifestyle and dietary characteristics of the study population.

Characteristic	Population value
Minutes of exercise/week prior to pandemic (median (interquartile range))	100 (180)
Minutes of exercise/week in the past week (median (min-max))	60 (146)
Weight change since start of coronavirus pandemic *n* (%)	
Yes, weight gain	929 (24.5)
Yes, weight loss	652 (17.2)
No	1226 (32.3)
Don’t know	990 (26.0)
Quantity of weight change among those reporting change	
Weight gained (median (interquartile range))	2 (1.5)
Weight lost (median (interquartile range))	−2 (2)
Vegan/Vegetarian *n* (%)	443 (11.7)
Takes nutrition supplements *n* (%)	1289 (33.9)
MedDiet Score (median (interquartile range))	9 (3)
MedDiet component questions	
Uses Olive Oil as Main Culinary Fat *n* (%)	2492 (65.6)
Eats poultry/white meat more than red meat *n* (%)	2475 (65.2)
Vegetable servings/day (median (interquartile range))	3 (3)
Fruit servings/day (median (interquartile range))	2 (2)
Butter/margarine/cream servings/day (median (interquartile range))	1 (2)
Sweetened beverages/day (median (interquartile range))	0 (1)
Whole grain servings/day (median (interquartile range))	2 (3)
Unsweetened dairy servings/day (median (interquartile range))	2 (2.5)
Red/processed meat servings/week (median (interquartile range))	1 (3)
Alcoholic beverages/week (median (interquartile range))	0 (3)
Legume servings/week (median (interquartile range))	2 (3)
Fish servings/week (median (interquartile range))	1 (2)
Nut servings/week (median (interquartile range))	1 (3)
Hummus/tahina servings/week (median (interquartile range))	1 (3)
Sweet baked goods servings/week (median (interquartile range))	3 (5)
Savory baked goods servings/week (median (interquartile range))	0 (2)
Salty snacks servings/week (median (interquartile range))	1 (3)
Change in diet quality *n* (%)	
Healthier prior to pandemic	2269 (59.8)
No difference	1009 (26.6)
Healthier with pandemic	495 (13.0)
No response	24 (0.6)

The Mediterranean Diet Score was calculated such that a given question receives a value of 1 if the criterion was met, and 0 if it was not, so the total score can range from 0 to 17 points.

Regarding physical activity, a 40% relative reduction in the time devoted to exercise was observed during the pandemic, declining from a median of 100–60 min per week; this difference was significant, *p* < 0.0001.

While no change in body weight was reported by almost 1/3 of respondents, almost 25% reported weight gain, and 17.2% reported weight loss. Weight change was a median 2 kg for both individuals who gained and those who lost weight.

Respondents were asked to rate the quality of their diets during the pandemic in relation to their usual diets. Most participants (59.8%) indicated that their pre-pandemic diet was healthier than their current diet. Slightly more than a quarter of participants did not detect a difference in their diets before vs. after the outbreak, while 13% felt their diet had improved during the pandemic. The reported change in body weight was significantly associated with the change in diet quality: among participants who gained weight, 87% indicated that their pre-outbreak diets were healthier than their current diets. By contrast, 46.3% of participants who reported no weight change and 47.9% of patients who reported weight loss indicated that their pre-pandemic diets were healthier, *p* < 0.0001. Indeed, the MedDiet score was significantly lower among individuals who felt their diets were healthier prior to the outbreak (median = 8, IQ = 3) vs. those who reported no difference in diet quality median = (9, IQ = 3) or those who reported that their diets had improved during the pandemic (median = 9, IQ = 3), *p* < 0.0001. MedDiet scores were also significantly lower among individuals who reported weight gain during the pandemic compared to those who lost weight (median = 8, IQ = 3 vs. median = 9, IQ = 4, *p* < 0.0001).

The MedDiet score was significantly, positively associated with participant characteristics including age: rho = 0.206, *p* < 0.0001; minutes of exercise per week prior to the outbreak: rho = 0.144, *p* < 0.0001; minutes of exercise during the outbreak: rho = 0.190, *p* < 0.0001. A significant inverse correlation was observed between the MedDiet score and body weight change, rho = −0.129, *p* < 0.0001. The MedDiet score did not differ by sex. A significant difference in the MedDiet score was detected across the languages in which the survey was completed (*p* < 0.0001); specifically, that the median (interquartile range) values were: Arabic: 9 (3); English 8 (3); French: 9 (3); Hebrew: 9 (3); Italian: 10 (4); Russian 6 (4); and Spanish: 10 (3).

### Anxiety

Measures of anxiety and emotional stress are shown in Table 3. The population score was a median 5 (IQ = 8), indicating mild anxiety levels. Isolation was rated on a 10-point scale at a median 3 (IQ = 4) in general, increasing to a median score of 4 (IQ = 5) after the crisis, *p* < 0.0001. While the median values for the number of people to which the respondent could turn to for urgent help appear identical before vs. during the coronavirus pandemic, the values, in fact, declined significantly from a mean of 4.2 ± 1.3 to 3.8 ± 1.4, *p* < 0.0001.

**Table 3:** Anxiety measures.

Characteristic	Population value
GAD-7 score (median (interquartile range))^a^	
Feeling nervous, anxious or on edge	1 (2)
Not being able to stop or control worrying	1 (1)
Worrying too much about different things	1 (2)
Trouble relaxing	1 (1)
Being so restless that it is hard to sit still	0 (1)
Becoming easily annoyed or irritable	1 (1)
Feeling afraid as if something terrible might happen	1 (1)
Total GAD-7 Score (median (interquartile range))	5 (8)
How isolated do you feel in general?^b^ (median (interquartile range)	3 (4)
How isolated do you feel in the current situation (during the pandemic)?^b^ (median (interquartile range)	4 (5)
In general, if you needed to borrow $20, or a ride to the doctor’s office, or any other urgent assistance, to how many people could you turn?^c^ (median (interquartile range)	5 (2)
In the current situation, if you needed to borrow $20, or a ride to the doctor’s office, or any other urgent assistance, to how many people could you turn?^c^ (median (interquartile range)	5 (2)

^a^The GAD-7 scale asks the respondent to refers to the 2 weeks prior to the survey. Each item on the scale can receive is scored as follows: 0 (not at all), 1 (several days), 2 (more than half of the days) 3 (nearly every day); thus, the total score can receive a value from 0 to 21, where a higher score indicates greater anxiety.

^b^The question about isolation requested the participant to respond on a 10-point Likert scale where 1 = very little and 10-very much.

^c^The question requested the participant to respond on a 7-point Likert scale where 0 = no one and 6 = more than 5 people.

### Associations between anxiety and MedDiet Score

The GAD-7 score was significantly, inversely correlated to the MedDiet score: rho = −0.159, *p* < 0.0001, indicating that the higher the anxiety score, the lower the diet quality score.

The GAD-7 score was significantly, positively associated with the following items of the MedDiet score: servings of butter, margarine or cream per day, rho = 0.170, *p* < 0.0001; number of sweetened beverages per day, rho = 0.109, *p* < 0.0001; servings of red or processed meat per week, rho = 0.079, *p* < 0.0001; number of alcoholic beverages per week, rho = 0.056, *p* < 0.0001; number of savory baked goods per week, rho = 0.196, *p* < 0.0001; and the number of salty snacks per week, rho = 0.267, *p* < 0.0001.

The GAD-7 score was significantly inversely associated with the following items of the MedDiet score: fish servings per week, rho = −0.114, *p* < 0.0001; servings of nuts per week, rho = −0.059, *p* < 0.0001; and hummus/tahini/refried beans, rho = −0.13, *p* < 0.0001.

Among individuals reporting that their diet was healthier prior to the coronavirus pandemic, the median total GAD-7 score was median = 6, (IQ = 9), compared to median = 3 (IQ = 6) among those who reported no change and median = 4 (IQ = 6) among those reporting an improvement in diet quality since the start of the outbreak. Similarly, the GAD-7 score was significantly higher in individuals who reported weight gain during the pandemic vs. those who reported weight loss: median = 7 (IQ = 9) vs. median = 5 (IQ = 9), *p* < 0.0001. Consistent with this, the GAD-7 score was significantly, positively associated with weight change, rho = 0.103, *p* < 0.0001. The GAD-7 score was positively associated with the number of minutes per week the respondent generally (prior to the outbreak) devoted to exercise (rho = 0.07, *p* < 0.0001), but was not significantly correlated to the number of minutes the respondent exercised weekly during the outbreak.

### Anxiety and respondent characteristics

The GAD-7 score was significantly, inversely associated with age: rho = −0.138, *p* < 0.0001. Table 4 displays the GAD-7 score by participant characteristics. Anxiety differed significantly by each of the characteristics tested. Women, and especially pregnant women, reported higher levels of anxiety. Anxiety also differed significantly by the language in which the survey was completed, such that those who completed the survey in English had the highest median scores, while those who completed the survey in Hebrew reported the lowest median scores. Individuals currently sick with coronavirus (*n* = 3) had the highest levels of anxiety, while those sick with any illness but coronavirus had the lowest anxiety levels. GAD-7 scores were also higher among individuals who are currently unemployed and those with fewer than 12 years of formal education.

**Table 4:** GAD-7 scores by study population characteristics.

Characteristic	GAD-7 score median (interquartile range)	*p* value
Sex		<0.0001
Female	6 (8)	
Male	3 (6)	
Reproductive status (Females only)		<0.0001
Neither pregnant nor 6 months post delivery	6 (8)	
6 months or less post delivery	6 (8.25)	
Currently pregnant	7 (9)	
Language		<0.0001
Arabic	6 (5)	
English	8 (10)	
French	6 (6.75)	
Hebrew	3 (5)	
Italian	6 (6)	
Russian	5 (8)	
Spanish	7 (7)	
Health status		<0.0001
Healthy	5 (8)	
Sick with coronavirus	15.5 (3)	
Suspected of coronavirus	8 (10)	
Recovering from coronavirus	6 (11)	
Sick but not with coronavirus	4 (7.5)	
Smoking status		<0.0001
Never smoked	5 (7)	
Former smoker	6 (10)	
Occasional smoker	5 (8)	
Smokes at least one cigarette/day	6 (9)	
Usual place of employment		<0.0001
Large/medium private sector company	6 (8)	
Small private sector company	5 (8)	
Public sector/non-profit	4 (7)	
Pensioner/unemployed	5 (8)	
Independent business owner/freelancer	5 (8)	
Other	3 (4)	
Work status during pandemic		<0.0001
Leave of absence with pay	5 (8)	
Leave of absence without pay	4 (7)	
Retired/Pensioner	4.5 (7)	
Still Working	5 (8)	
Unemployed	7 (9)	
Other	3 (6)	
Education		<0.0001
Fewer than 12 years	7 (6.75)	
High school diploma/matriculation	5 (8)	
Professional license (technician, tradesperson, etc.)	6 (9)	
Bachelor’s degree	5 (8)	
Master’s degree or higher	5 (6)	
Other	4 (12)	

Table 5 presents the multivariable logistic regression model of moderate to high anxiety (GAD-7 score of 10 or greater). The following variables were entered into the regression model: age, sex, the language in which the questionnaire was completed, duration of physical activity prior to the COVID-19 outbreak (minutes/week), education, change in diet pattern, smoking status (current smoker yes/no) and MedDiet score. The final model was arrived at using a backward, stepwise approach and did not include minutes per week of physical activity or current smoking. The model was significant and correctly classified 75.6% of study participants for moderate anxiety or more severe. As can be seen, women were more than twice as likely as men to experience at least moderate anxiety, as were individuals who reported that their current diets were less healthy than their diets prior to the COVID-19 pandemic. Language was significantly associated with anxiety, such that completing the survey in Arabic, Hebrew, Italian or Russian (vs. English) significantly reduced the odds of moderate to severe anxiety. Age also significantly reduced the risk of moderate to severe anxiety. Importantly, every one-point increase in the MedDiet score reduced risk of moderate to severe anxiety by a relative 8% (5–11%).

**Table 5:** Multivariable logistic regression model of moderate to severe anxiety (GAD-7 score ≥ 10).

Variable	Odds ratio	95% confidence interval	*p* value
MedDiet Score	0.92	0.89–0.95	<0.001
Age (years)	0.99	0.98–0.99	<0.001
Sex (F = 1)	2.22	1.81–2.74	<0.001
Education (college degree = 1)	0.84	0.70–1.00	0.052
Language			
English			<0.001
Arabic = 1	0.34	0.25–0.47	<0.001
French = 2	0.59	0.23–1.53	0.278
Hebrew = 3	0.15	0.12–0.18	<0.001
Italian = 4	0.49	0.30–0.78	0.003
Russian = 5	0.0.48	0.23–0.97	0.04
Spanish	0.70	0.39–1.25	0.23
Change in diet (diet worse = 1)	1.61	1.24–2.09	<0.001
Constant	0.93		0.738

Female sex (vs. male) was the indicator variables for sex; any college degree (bachelor’s degree or higher = 1 (vs. any other response) and was the indicator variable for education; Spanish was the indicator variable for language (vs. any other language); reporting that the current diet was worse than the pre-pandemic diet = 1 (vs. no change or improvement in diet = 0) and was the indicator for change in diet.

## Discussion

The present study indicates that diet quality was inversely associated with moderate to severe anxiety during the 2020 COVID-19 pandemic. Specifically, in our model, every one-point increase in the MedDiet score was associated with an almost 7% decrease in the odds of moderate to severe anxiety. More than 50% of study participants had a GAD-7 score consistent with at least mild anxiety. This rate is considerably higher than the prevalence of anxiety in the general population, which is estimated to be 1.9–5.1% [23]. However, it must be pointed out that our study population is a convenience sample, and thus it is conceivable that particularly anxious individuals chose to participate in the survey.

Increased MedDiet score was associated with reduced odds of moderate to severe anxiety, while a decline in diet quality during the outbreak compared to the pre-pandemic diet increased risk for this outcome, even after controlling for key explanatory variables. In univariate analysis, weight gain was associated with an increased median GAD-7 score. A number of mechanisms have been proposed to explain how diet may modulate anxiety. A high fat diet has been associated with increased corticosterone levels and increased levels of circulating inflammatory cytokines and disruption of intracellular cascades involved in synaptic plasticity [24]. A high refined carbohydrate diet has been shown to cause microglial cells to release pro-inflammatory cytokines, increasing nitric oxide production, which appears to promote anxiety in humans [25].

It has been shown that metabolic status (including obesity), age and sex influence the clinical severity of COVID-19 [26, 27]. This suggests that short, abrupt increases in energy intake coupled with reduced physical activity could produce adverse health consequences in a limited time frame. Indeed, these acute changes have been associated with increased total body fat and particularly abdominal fat, possibly driven by insulin resistance and increased circulating inflammatory cytokines [28]. Thus, adequate control of metabolic disorders could be important to reduce the risk of severe COVID-19.

Uneven COVID-19 mortality rates across Europe may be partially explained by differences in nutrition. Specifically, diets high in antioxidants or anti-angiotensin convertin enzyme (ACE) activity such as uncooked or fermented cabbage, are more frequently consumed European countries with relatively low COVID-19 death rates [29].

Poor diet quality (which may foreshadow obesity) age and sex were associated with anxiety in the present study. Age reduced risk for moderate to severe anxiety in study participants. An inverse association between age and anxiety symptoms has been reported previously; specifically, the longitudinal trajectory of anxiety is downward from middle age through the 7th decade of life, with a modest rebound thereafter that tapers off [30]. Female sex was associated with increased anxiety, a finding consistent with prior reports. The increase in generalized anxiety in girls begins in mid-adolescence, and the lifetime prevalence of this condition in women is almost twice that of men [31].

A higher MedDiet score was also associated with less self-reported weight gain and an improved diet pattern during the pandemic. These findings may have implications for public health interventions. For example, online programs promoting adoption and/or maintenance of healthy lifestyle habits, particularly diet, coupled with stress reduction techniques, might benefit populations during any future pandemic.

It is also interesting that the language in which the survey was completed influenced GAD-7 scores. Completing the survey in English was associated with increased risk for moderate-severe anxiety relative to Arabic, Hebrew, French, and Russian. Cultural differences in anxiety have been reported [32]. In the setting of the pandemic, differences could be due to cross-cultural differences, or they could be due to differences in the severity of the pandemic and/or public health messaging in locations where surveys were answered in English.

Findings herein must be considered in the framework of study limitations. The data are cross-sectional. Associations between diet quality and anxiety are correlational only. Further, many of the correlations were relatively weak though significant, likely due to large sample size. Because the exposure (diet quality) and outcome (anxiety score) were measured simultaneously, the temporal order cannot be ascertained and, as such, causality cannot be inferred.

The present study nevertheless reports on a very large, multinational group of respondents during the rapid contagion period of the COVID-19 pandemic. The study power was adequate to permit identification of population behaviors during a global pandemic and observes an inverse association between diet quality and anxiety level. Unfortunately, it is not possible to determine whether anxiety triggers unhealthy eating patterns, or unhealthy eating patterns reinforce anxiety. Perhaps the association is bi-directional.

This large, internationally distributed online survey conducted in a variety of widely-spoken languages detects associations between anxiety levels and dietary patterns, albeit in a self-selected sample. Poor diet quality was associated with weight gain, which could contribute to obesity; however, the cross-sectional study design precludes any inference about causality. Preventive policy aimed at diet quality and anxiety should be directly tested to determine if it can reduce obesity-associated adverse COVID-19 outcomes.
